# A Comprehensive Calibration Method for a Star Tracker and Gyroscope Units Integrated System

**DOI:** 10.3390/s18093106

**Published:** 2018-09-14

**Authors:** Wenfeng Tan, Dongkai Dai, Wei Wu, Xingshu Wang, Shiqiao Qin

**Affiliations:** College of Opto-Electronic Science and Engineering, National University of Defense Technology, Changsha 410073, China; tanwenfeng08@nudt.edu.cn (W.T.); weiwunudt@gmail.com (W.W.); wangxingshu@sohu.com (X.W.); sqqin8@nudt.edu.cn (S.Q.)

**Keywords:** star tracker, gyroscope units, comprehensive calibration, Kalman filter

## Abstract

The integration of a star tracker and gyroscope units (GUs) can take full advantage of the benefits of each, and provide continuous and accurate attitude information with a high update rate. The systematic error calibration of the integrated system is a crucial step to guarantee its attitude accuracy. In this paper, a comprehensive calibration method for the star tracker and GUs integrated system is proposed from a global perspective. Firstly, the observation model of the predicted star centroid error (PSCE) with respect to the systematic errors including the star tracker intrinsic parameter errors, GUs errors and fixed angle errors is accurately established. Then, the systematic errors are modeled by a series of differential equations, based on which the state-space model is established. Finally, the systematic errors are decoupled and estimated by a Kalman filter according to the established state-space model and observation model. The coupling between the errors of the principal point and subcomponents of the fixed angles (i.e., $\Psi_{x}$ and $\Psi_{y}$) is analysed. Both simulations and experiments indicate that the proposed method is effective at estimating the systematic errors of the star tracker and GUs integrated system with high accuracy and robustness with respect to different star centroid accuracies and gyroscope noise levels.

## 1. Introduction

A star tracker, which can provide high-accuracy attitude information with respect to an inertial frame [1,2,3,4], has been widely used in the fields of attitude determination, guidance and navigation [5,6,7]. However, the attitude update rate of the star tracker is seriously restricted by the time of pixel data transmission and processing [8], which will limit its application under highly dynamic conditions. The shortage of a star tracker can be overcome by combining it with three-axis gyroscope units (GUs). The GUs consist of three orthogonally assembled gyroscopes and can provide continuous attitude information in the inertial frame with high update rate. Although the attitude error of the GUs accumulates over time due to the gyroscope and initialization errors, it can be well compensated by the star tracker measurements. Hence, the star tracker and GUs integrated system can take advantage of the benefits of each and has the potential to provide continuous, highly accurate attitude information with high update rate.

The accuracy of the star tracker and GUs integrated system significantly depends on the calibration accuracy of the systematic errors including the star tracker intrinsic parameter errors, GUs errors and fixed angle errors between them. Traditional methods mainly focus on the calibration of the single star tracker. A typical calibration method is conducted by simulating starlight coming from different directions with an expensive precision turntable and a highly accurate star simulator in the laboratory [9,10,11,12,13,14]. Laboratory calibration is generally more precise and has a more convenient calibration process [14]. Although the star tracker can be accurately calibrated in the laboratory, these parameters may change due to the difference between laboratory and on-orbit enviroments. Moreover, many factors, such as vibrations during launch, component aging and variable environments during operation, alter the systematic errors and bring about mismatches between default parameters and on-orbit ones, leading to degraded attitude accuracy [15]. Hence, on-orbit calibration is essential for guaranteeing the performance of the star tracker during service. The on-orbit calibration method based on the invariance of the interstar angle [16] was first proposed by Samaan et al. Due to the attitude-independent characteristics, this method is the most widely used on-orbit calibration approach. It was developed by many researchers in terms of the parameter estimation algorithms and improvements to the measurement model. Singla et al. [17] adopted the combination of Least Squares and the Kalman filter to estimate the principal point and the focal length of the star tracker. Liu et al. [18] proposed a modified Least Squares iteration algorithm combining Kalman filter with a two-step procedure for on-orbit calibration of a star tracker. The principal point and focal length were achieved at first, then the lens distortion coefficients were estimated using the solutions of the first step. Zhang et al. [15] proposed a self-initialization on-orbit calibration method combining the back propagation neural network and the unscented Kalman filter. In addition, Li et al. [19] proposed a star tracker on-orbit calibration method based on vector pattern match. Wang et al. [20] proposed an on-orbit attitude-dependent calibration method for a navigation camera, whose attitudes were provided by a simulated attitude determination and control system with accuracy 3” (standard deviation). All methods mentioned above can achieve the parameter calibration of a single star tracker.

It should be noted that the performance of a star tracker and GUs integrated system depends not only on the calibration accuracy of the star tracker but also on the GUs errors and fixed angle errors. Since aforementioned methods focus on the calibration of a single star tracker, the GUs errors and fixed angle errors need to be calibrated individually. In this paper, we propose a comprehensive calibration method for a star tracker and GUs integrated system from a global perspective. Since the predicted star centroid error (PSCE) is induced by the systematic errors including the star tracker intrinsic parameter errors, GUs errors and fixed angle errors, it is possible to estimate these errors by observing the PSCE in the image plane. A Kalman filter can be used to estimate these systematic errors optimally with the established state-space model and observation model, therefore the performance of the integrated system can be improved.

This paper is organized as follows. In Section 2, the theory of this comprehensive calibration method for the star tracker and GUs integrated system is explained. First, the physical measurement model is deduced. Then, the state-space model and the observation model for estimations of the systematic errors are established. Simulation and experimental results are presented in Section 3 and Section 4 respectively. Finally, conclusions are presented in Section 5.

## 2. Theory of the Comprehensive Calibration Method for the Star Tracker and GUs Integrated System

### 2.1. Reference Coordinate System Definition

The coordinate systems used in this paper are defined as follows:

The inertial coordinate system (denoted by *i* in subscript) coincides with the International Celestial Reference System recommended by the IAU Working Group on Nomenclature for Fundamental Astronomy. It has no intrinsic orientation but is aligned close to the mean equator and dynamical equinox of J2000.0. Its orientation is independent of epoch, ecliptic or equator and is defined by a list of adopted coordinates of extragalactic sources [21].

**Figure 1:**
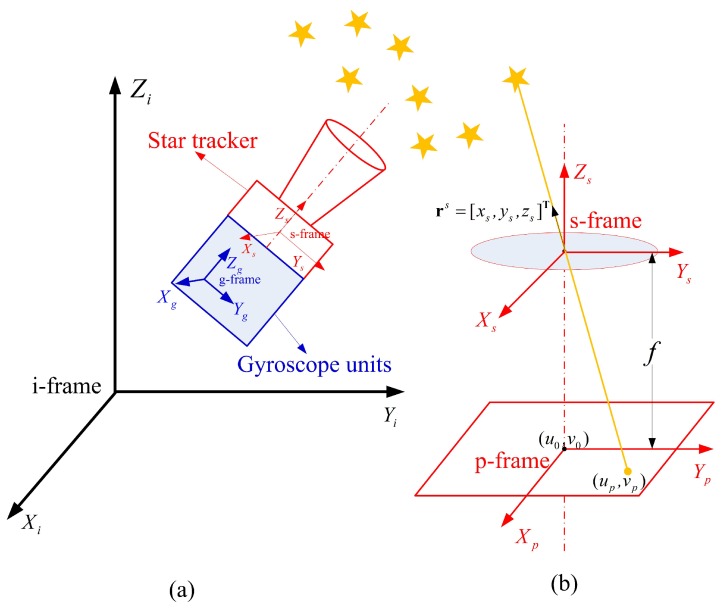
Definition of coordinate systems. Part (**b**) shows the enlarged details of the star tracker in Part (**a**).

The star tracker coordinate system (denoted by *s* in subscript) has its origin at the center of the star tracker optical system. The $X_{s}$ and $Y_{s}$ axes are parallel to the two vertical edges of the detector plane, respectively. The $Z_{s}$ axis is along the boresight of the star tracker and the three axes satisfy the right-hand rule [22] as shown in Figure 1a.

The gyroscope units coordinate system (denoted by *g* in subscript) has its $X_{g}$, $Y_{g}$ and $Z_{g}$ axes consistent with the three mutually orthogonal sensitive axes of the gyroscope units as shown in Figure 1a [23].

The image plane coordinate system (denoted by *p* in subscript) is a two-dimensional rectangular plane coordinate system with its origin at the detector center, and its $X_{p}$ and $Y_{p}$ axes are parallel to the two vertical edges of the detector plane respectively as shown in Figure 1b [23].

### 2.2. Estimation of Systematic Errors by Observing the Predicted Star Centroid Error in the Image Plane

The procedure for estimations of systematic errors of the star tracker and GUs integrated system is as follows: The predicted star centroid (PSC) can be calculated based on the GUs attitude and fixed angles between the star tracker and GUs. Simultaneously, the extracted star centroid (ESC) can be obtained by extracting the star centroid from the real star image collected by the star tracker. Considering an error-free situation, the PSC equals to ESC. However, the systematic errors including the star tracker intrinsic parameter errors, GUs errors and fixed angle errors will introduce the predicted star centroid error (PSCE), and cause the difference between the PSC and ESC. Since the PSCE is induced by the systematic errors of the star tracker and GUs integrated system, these errors can be estimated by observing the PSCE.

#### 2.2.1. Star Centroid Prediction Based on GUs

With the star right ascension and declination (α,δ) provided by the star catalog, the star vector $r^{i}$ in inertial frame *i* can be calculated by:

(1) $$r^{i}={\left[cos\delta cos\alpha ,cos\delta sin\alpha ,sin\delta \right]}^{T}.$$

The star vector in inertial frame *i* can be transformed to the star tracker frame *s* by:

(2) $$r^{s}=C_{g}^{s}C_{i}^{g}r^{i},$$

where $C_{i}^{g}$ is the attitude matrix from frame *i* to gyroscope units frame *g* calculated by an attitude updating algorithm [24] with angular increments sensed by the GUs. $C_{g}^{s}$ is the transformation matrix from frame *g* to frame *s* and is called the installation matrix.

Since the star tracker can be considered as a pinhole imaging model as shown in Figure 1b, the PSC $\left(u_{p},v_{p}\right)$ in the image plane can be expressed as:

(3) $$\left\{\begin{array}{c} u_{p}=u_{0}-f\frac{x_{s}}{z_{s}}+u_{d}\\ v_{p}=v_{0}-f\frac{y_{s}}{z_{s}}+v_{d} \end{array}\right.,$$

where $\left(u_{0},v_{0}\right)$ is the principal point, *f* is the focal length of the lens, $\left(u_{d},v_{d}\right)$ is the lens distortion in the two directions of the image plane respectively, and $r^{s}={\left[x_{s},y_{s},z_{s}\right]}^{T}$ is the star vector in frame *s*. Here, we adopt the following model to describe the lens distortion [25]:

(4) $$\left\{\begin{array}{c} u_{d}=\left(u-u_{0}\right)\left(k_{1}r^{2}+k_{2}r^{4}\right)+p_{1}\left[r^{2}+2{\left(u-u_{0}\right)}^{2}\right]+2p_{2}\left(u-u_{0}\right)\left(v-v_{0}\right)\\ v_{d}=\left(v-v_{0}\right)\left(k_{1}r^{2}+k_{2}r^{4}\right)+p_{2}\left[r^{2}+2{\left(v-v_{0}\right)}^{2}\right]+2p_{1}\left(u-u_{0}\right)\left(v-v_{0}\right) \end{array}\right.,$$

where $k_{1},k_{2}$ are coefficients for radial distortion, $p_{1},p_{2}$ are coefficients for tangential distortion, and $r=\sqrt{{\left(u-u_{0}\right)}^{2}+{\left(v-v_{0}\right)}^{2}}$.

Equations (1)–(4) show the star centroid prediction process based on the GUs.

On the other hand, the ESC $\left({\hat{u}}_{e},{\hat{v}}_{e}\right)$ deviates from its true position (u,v) due to the star extraction errors, which can be expressed as:

(5) $$\left\{\begin{array}{c} {\hat{u}}_{e}=u+w_{u}\\ {\hat{v}}_{e}=v+w_{v} \end{array}\right.,$$

where $w_{u},w_{v}$ are the star extraction errors in *x* and *y* directions of frame *p* respectively. Considering that (u,v) can be expressed with the star tracker imaging model, Equation (5) can be further expressed as:

(6) $$\left\{\begin{array}{c} {\hat{u}}_{e}=u_{0}-f\frac{x_{s}}{z_{s}}+u_{d}+w_{u}\\ {\hat{v}}_{e}=v_{0}-f\frac{y_{s}}{z_{s}}+v_{d}+w_{v} \end{array}\right..$$

According to Equations (3) and (6), provided with accurate parameters of the integrated system, the difference between the PSC and ESC only lies in the star extraction errors $w_{u},w_{v}$. However, the systematic errors (i.e., the star tracker intrinsic parameter errors, GUs errors and fixed angle errors) will also cause the deviation of PSC from the ESC. Next, we will discuss the PSCE induced by these systematic errors.

#### 2.2.2. Predicted Star Centroid Error Induced by Systematic Errors

The GUs can sense the three-dimensional angular increments and provide the attitude of the GUs in frame *i* (i.e., $C_{i}^{g}$). Generally, its accuracy degrades with time due to the cumulative error of the gyroscopes. Let ${\hat{C}}_{i}^{g}$ denote the actual attitude matrix contaminated by gyroscope errors [24]:

(7) $${\hat{C}}_{i}^{g}=C_{i}^{g}C_{i^{\prime }}^{i}\approx C_{i}^{g}\left(I_{3\times 3}+\left[\begin{array}{ccc} 0 & -\phi_{z} & \phi_{y}\\ \phi_{z} & 0 & -\phi_{x}\\ -\phi_{y} & \phi_{x} & 0 \end{array}\right]\right)=C_{i}^{g}\left(I_{3\times 3}+\left[\phi \times \right]\right),$$

where $I_{3\times 3}$ represents the identity matrix of size 3×3, ϕ is the attitude error in the form of Euler angle in frame *i*, and frame $i^{\prime }$ represents the computed inertial coordinate system contaminated by gyroscope errors.

Due to the inaccuracy of the installation matrix, the actual transformation matrix from frame *g* to frame *s* ${\hat{C}}_{g}^{s}$ can be expressed as [24]:

(8) $${\hat{C}}_{g}^{s}=C_{s}^{s^{\prime }}C_{g}^{s}\approx \left(I_{3\times 3}+\left[\Psi \times \right]\right)C_{g}^{s},$$

where Ψ is the fixed angle vector error in frame *s*, $\left[\Psi \times \right]$ is the skew symmetric form of Ψ with similar expression to [ϕ×] in Equation (7), and frame $s^{\prime }$ represents the computed star tracker coordinate system contaminated by fixed angle errors. According to Equations (7) and (8), the star vector transformation from frame *i* to frame *s* expressed by Equation (2) should be rewritten as:

(9) $${\hat{r}}^{s}={\hat{C}}_{g}^{s}{\hat{C}}_{i}^{g}r^{i}=\left(I_{3\times 3}+\left[\Psi \times \right]\right)C_{g}^{s}C_{i}^{g}\left(I_{3\times 3}+\left[\phi \times \right]\right)r^{i},$$

where ${\hat{r}}^{s}={\left[{\hat{x}}_{s},{\hat{y}}_{s},{\hat{z}}_{s}\right]}^{T}$ is the predicted star vector contaminated by systematic errors in frame *s*.

Given the star vector ${\hat{r}}^{s}$ in frame *s*, the PSC in frame *p* can be calculated with the star tracker imaging model given by Equation (3). Considering the intrinsic parameter errors $\delta u_{0},\delta v_{0},\delta f,\delta u_{d},\delta v_{d}$ of the star tracker, Equation (3) should be rewritten as:

(10) $$\left\{\begin{array}{c} {\hat{u}}_{p}={\hat{u}}_{0}-\hat{f}\frac{{\hat{x}}_{s}}{{\hat{z}}_{s}}+{\hat{u}}_{d}\\ {\hat{v}}_{p}={\hat{v}}_{0}-\hat{f}\frac{{\hat{y}}_{s}}{{\hat{z}}_{s}}+{\hat{v}}_{d} \end{array}\right.,$$

where (${\hat{u}}_{p},{\hat{v}}_{p}$) is the PSC in the image plane, $\left({\hat{u}}_{0},{\hat{v}}_{0}\right)$ is the initial value of principal point satisfying ${\hat{u}}_{0}=u_{0}+\delta u_{0}$ and ${\hat{v}}_{0}=v_{0}+\delta v_{0}$, $\hat{f}$ is the initial value of focal length satisfying $\hat{f}=f+\delta f$, and $\left({\hat{u}}_{d},{\hat{v}}_{d}\right)$ is the initial value of lens distortion satisfying ${\hat{u}}_{d}=u_{d}+\delta u_{d}$ and ${\hat{v}}_{d}=v_{d}+\delta v_{d}$.

Subtracting Equation (6) from Equation (10) and neglecting the higher order error terms, we have the PSCE (δu,δv) as follows:

(11) $$\left\{\begin{array}{c} \delta u={\hat{u}}_{p}-{\hat{u}}_{e}=\delta u_{0}-\delta f\frac{x_{s}}{z_{s}}-f\frac{\delta x_{s}}{z_{s}}+f\frac{x_{s}}{z_{s}^{2}}\delta z_{s}+\delta u_{d}-w_{u}\\ \delta v={\hat{v}}_{p}-{\hat{v}}_{e}=\delta v_{0}-\delta f\frac{y_{s}}{z_{s}}-f\frac{\delta y_{s}}{z_{s}}+f\frac{y_{s}}{z_{s}^{2}}\delta z_{s}+\delta v_{d}-w_{v} \end{array}\right.,$$

where $\delta r^{s}={\left[\delta x_{s},\delta y_{s},\delta z_{s}\right]}^{T}$ is the star vector error in frame *s*. According to Equations (2) and (9), it can be expressed as:

(12) $$\begin{array}{cc} \delta r^{s} & ={\hat{r}}^{s}-r^{s}\\ & =\left[\Psi \times \right]C_{g}^{s}C_{i}^{g}r^{i}+C_{g}^{s}C_{i}^{g}\left[\phi \times \right]r^{i}+\left[\Psi \times \right]C_{g}^{s}C_{i}^{g}\left[\phi \times \right]r^{i}. \end{array}$$

Neglect the higher order error term (i.e., the last term of Equation (12)), and Equation (12) can be simplified as:

(13) $$\begin{array}{cc} \delta r^{s} & \approx \left[\Psi \times \right]C_{g}^{s}C_{i}^{g}r^{i}+C_{g}^{s}C_{i}^{g}\left[\phi \times \right]r^{i}\\ & =-\left[r^{s}\times \right]\Psi -C_{g}^{s}C_{i}^{g}\left[r^{i}\times \right]\phi . \end{array}$$

Let $A=-\left[r^{s}\times \right]$ and $B=-C_{g}^{s}C_{i}^{g}\left[r^{i}\times \right]$, and Equation (13) can be rewritten as:

(14) $$\delta r^{s}={\left[\delta x_{s},\delta y_{s},\delta z_{s}\right]}^{T}=A\Psi +B\phi .$$

Substituting Equation (14) into Equation (11) results in the final expression of the PSCE induced by systematic errors:

(15) $$\left\{\begin{array}{c} \delta u=\delta u_{0}-\delta f\frac{x_{s}}{z_{s}}+\left(-\frac{f}{z_{s}}A_{1}+f\frac{x_{s}}{z_{s}^{2}}A_{3}\right)\Psi +\left(-\frac{f}{z_{s}}B_{1}+f\frac{x_{s}}{z_{s}^{2}}B_{3}\right)\phi +\delta u_{d}-w_{u}\\ \delta v=\delta v_{0}-\delta f\frac{y_{s}}{z_{s}}+\left(-\frac{f}{z_{s}}A_{2}+f\frac{y_{s}}{z_{s}^{2}}A_{3}\right)\Psi +\left(-\frac{f}{z_{s}}B_{2}+f\frac{y_{s}}{z_{s}^{2}}B_{3}\right)\phi +\delta v_{d}-w_{v} \end{array}\right.,$$

where $A_{i}$ (i=1,2,3) is the *i*-th row of the matrix *A*, and the same definition applies to $B_{i}$. The lens distortion error ($\delta u_{d},\delta v_{d}$) can be expressed as:

(16) $$\left[\begin{array}{c} \delta u_{d}\\ \delta v_{d} \end{array}\right]=\left[\begin{array}{cccccc} \frac{\partial u_{d}}{\partial u_{0}} & \frac{\partial u_{d}}{\partial v_{0}} & \frac{\partial u_{d}}{\partial k_{1}} & \frac{\partial u_{d}}{\partial k_{2}} & \frac{\partial u_{d}}{\partial p_{1}} & \frac{\partial u_{d}}{\partial p_{2}}\\ \frac{\partial v_{d}}{\partial u_{0}} & \frac{\partial v_{d}}{\partial v_{0}} & \frac{\partial v_{d}}{\partial k_{1}} & \frac{\partial v_{d}}{\partial k_{2}} & \frac{\partial v_{d}}{\partial p_{1}} & \frac{\partial v_{d}}{\partial p_{2}} \end{array}\right]\left[\begin{array}{c} \delta u_{0}\\ \delta v_{0}\\ \delta k_{1}\\ \delta k_{2}\\ \delta p_{1}\\ \delta p_{2} \end{array}\right].$$

Hence the model of the PSCE induced by the systematic errors is established by Equations (15) and (16).

#### 2.2.3. Optimal Estimation of Systematic Errors

In Section 2.2.2, we established the linear model of observations δu,δv relating to the systematic errors including the star tracker intrinsic parameter errors, GUs errors and fixed angle errors. These parameters can be easily estimated with a Kalman filter by establishing the state-space model and the observation model.

The dynamics of the attitude error ϕ in frame *i* are given by Equation (17)

(17) $$\dot{\phi }=-C_{g}^{i}\varepsilon^{g},$$

where the gyroscope bias $\varepsilon^{g}$ can be modeled as random constants with:

(18) $${\dot{\varepsilon }}^{g}=0.$$

The fixed angle vector error can also be modeled as constants:

(19) $$\dot{\Psi }=0.$$

The star tracker intrinsic parameter errors can be modeled as:

(20) $$\left\{\begin{array}{c} \dot{\delta u_{0}}=0,\dot{\delta v_{0}}=0,\delta \dot{f}=0\\ \dot{\delta k_{1}}=0,\dot{\delta k_{2}}=0,\dot{\delta p_{1}}=0,\dot{\delta p_{2}}=0 \end{array}\right..$$

The state-space vector is given by:

(21) $$X={\left[\phi^{T},{\varepsilon^{g}}^{T},\Psi^{T},\delta u_{0},\delta v_{0},\delta f,\delta k_{1},\delta k_{2},\delta p_{1},\delta p_{2}\right]}^{T}.$$

According to Equations (17)–(21), the corresponding state-space model can be written as:

(22) $$\frac{d}{dt}X=\left[\begin{array}{cccc} 0_{3\times 3} & -C_{g}^{i} & 0_{3\times 3} & 0_{3\times 7}\\ 0_{13\times 3} & 0_{13\times 3} & 0_{13\times 3} & 0_{13\times 7} \end{array}\right]X+\left[\begin{array}{c} -C_{g}^{i}\\ 0_{13\times 3} \end{array}\right]w_{g},$$

where $w_{g}$ is the random noise of the gyroscopes, $0_{i\times j}$ is the zero-element matrix with size i×j.

According to Equations (15) and (16), the observation model for each star can be expressed as:

(23) y=hX+v,

where $y={\left[\delta u,\delta v\right]}^{T}$, and v is the star extraction noise vector. The matrix h is given by:

(24) $$h=\left[\begin{array}{ccccccc} -\frac{f}{z_{s}}B_{1}+f\frac{x_{s}}{z_{s}^{2}}B_{3} & 0_{1\times 3} & -\frac{f}{z_{s}}A_{1}+f\frac{x_{s}}{z_{s}^{2}}A_{3} & 1+\frac{\partial u_{d}}{\partial u_{0}} & \frac{\partial u_{d}}{\partial v_{0}} & -\frac{x_{s}}{z_{s}} & F_{1\times 4}^{u}\\ -\frac{f}{z_{s}}B_{2}+f\frac{y_{s}}{z_{s}^{2}}B_{3} & 0_{1\times 3} & -\frac{f}{z_{s}}A_{2}+f\frac{y_{s}}{z_{s}^{2}}A_{3} & \frac{\partial v_{d}}{\partial u_{0}} & 1+\frac{\partial v_{d}}{\partial v_{0}} & -\frac{y_{s}}{z_{s}} & F_{1\times 4}^{v} \end{array}\right],$$

where $F_{1\times 4}^{u}=\left[\begin{array}{cccc} \frac{\partial u_{d}}{\partial k_{1}} & \frac{\partial u_{d}}{\partial k_{2}} & \frac{\partial u_{d}}{\partial p_{1}} & \frac{\partial u_{d}}{\partial p_{2}} \end{array}\right]$ and $F_{1\times 4}^{v}=\left[\begin{array}{cccc} \frac{\partial v_{d}}{\partial k_{1}} & \frac{\partial v_{d}}{\partial k_{2}} & \frac{\partial v_{d}}{\partial p_{1}} & \frac{\partial v_{d}}{\partial p_{2}} \end{array}\right]$.

Equation (23) is deduced for each star across the star tracker’s field of view. For a star image with *n* stars, it can be extended as:

(25) Y=HX+V,

where

(26) $$Y=\left[\begin{array}{c} y_{1}\\ y_{2}\\ \vdots \\ y_{n} \end{array}\right],H=\left[\begin{array}{c} h_{1}\\ h_{2}\\ \vdots \\ h_{n} \end{array}\right],V=\left[\begin{array}{c} v_{1}\\ v_{2}\\ \vdots \\ v_{n} \end{array}\right],$$

and j=1,2,⋯,n denotes the *j*-th star of the star image.

Hence, the state-space model (i.e., Equation (22)) and the observation model (i.e., Equation (25)) are established. The state-space vector can be easily estimated by a Kalman filter [26] and the optimal estimations of overall systematic errors $\tilde{\phi },{\tilde{\varepsilon }}^{g},\tilde{\Psi },{\tilde{u}}_{0},{\tilde{v}}_{0},\tilde{f},{\tilde{k}}_{1},{\tilde{k}}_{2},{\tilde{p}}_{1},{\tilde{p}}_{2}$ can be acquired.

## 3. Simulation

**Figure 2:**
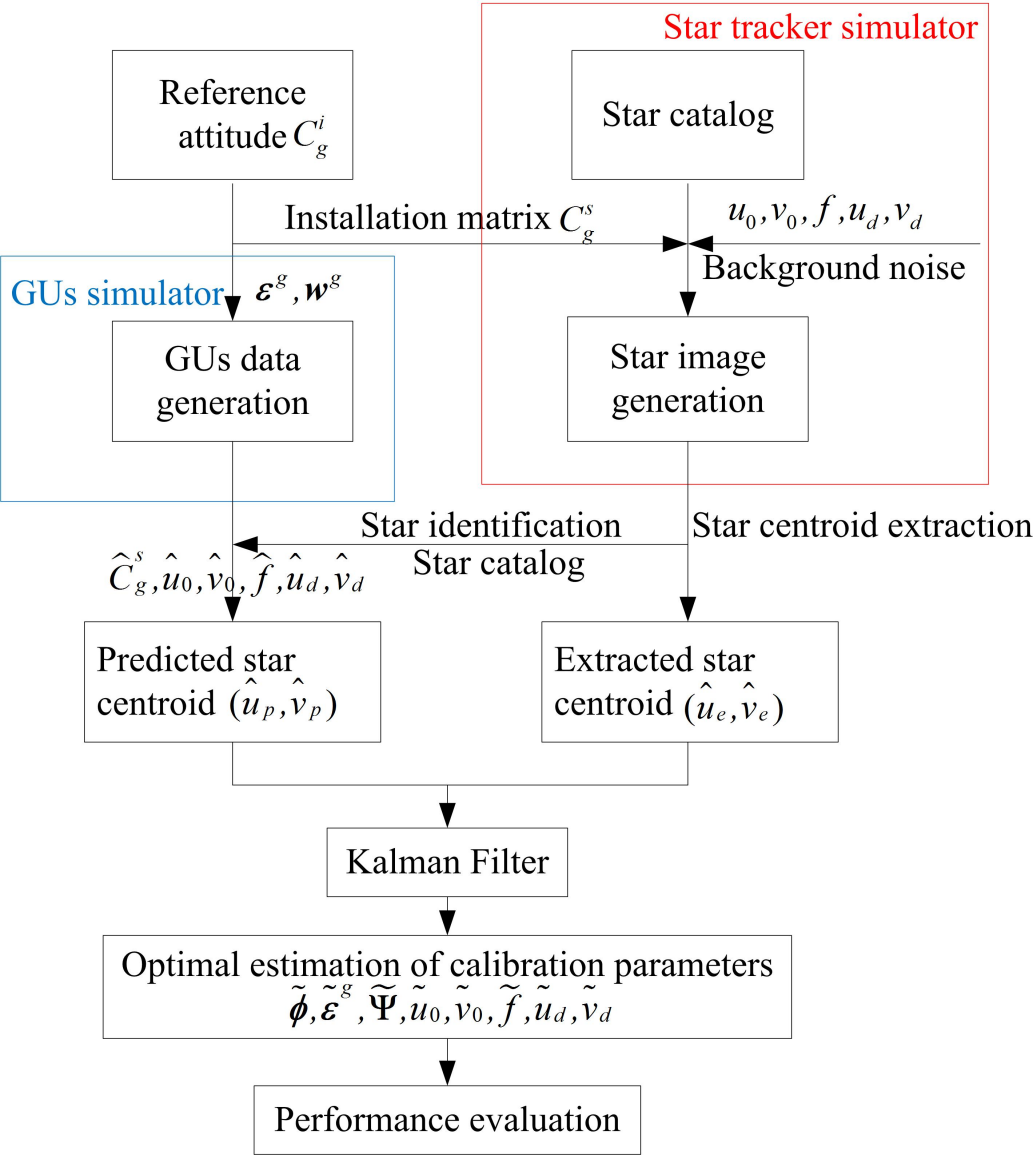
Simulation flow chart.

Several groups of simulations are designed to verify the feasibility of the proposed method. The procedure for each simulation is given in Figure 2. The star tracker simulator can generate corresponding star images with true model parameters $C_{g}^{s},u_{0},v_{0},f,u_{d},v_{d}$ and the preset reference attitudes $C_{g}^{i}$. The ESC $\left({\hat{u}}_{e},{\hat{v}}_{e}\right)$ can be obtained with the centroid extraction algorithm. Simultaneously, the GUs simulator can generate the three-axis angular rate of the GUs, which is sequentially used to calculate the PSC $\left({\hat{u}}_{p},{\hat{v}}_{p}\right)$ with parameters ${\hat{C}}_{g}^{s},{\hat{u}}_{0},{\hat{v}}_{0},\hat{f},{\hat{u}}_{d},{\hat{v}}_{d}$ contaminated by errors. Then the systematic errors are estimated with the proposed method with a Kalman Filter. The performance evaluation is conducted by: (1) comparing the estimated model parameters with preset true values; (2) comparing the estimated attitudes with preset reference attitudes; (3) calculating the reprojection error with estimated parameters.

### 3.1. Simulation Using Proposed Method

A typical star tracker and gyroscopes of navigation grade are used for this simulation with performance specifications listed in Table 1. The rotation sequence of the integrated system for calibration is shown in Table 2 and will be repeated during the whole calibration process.

**Figure 3:**
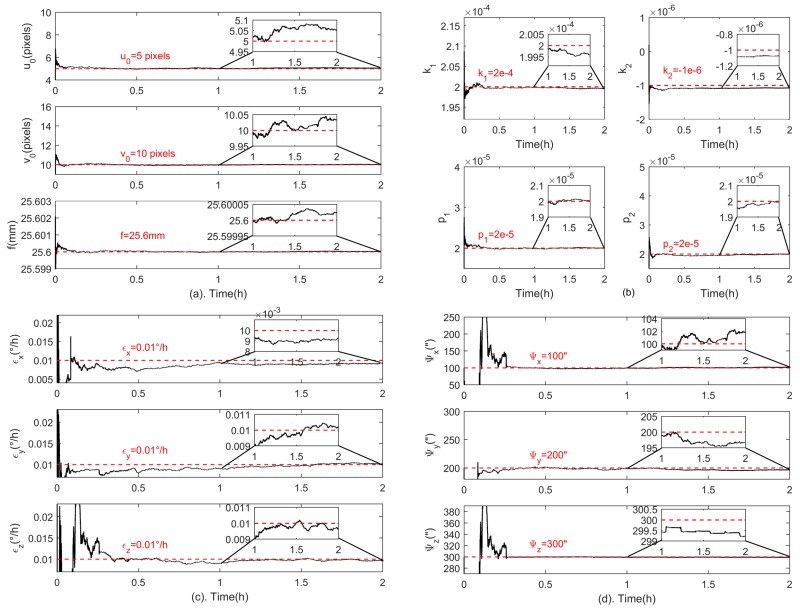
Estimation results of all systematic errors in the simulation. (**a**) shows estimations for the principal point $\left(u_{0},v_{0}\right)$ and the focal length *f*, (**b**) shows estimations for lens distortion coefficients $k_{1},k_{2},p_{1},p_{2}$, (**c**,**d**) show estimations for the gyroscope bias and fixed angle errors respectively.

**Figure 4:**
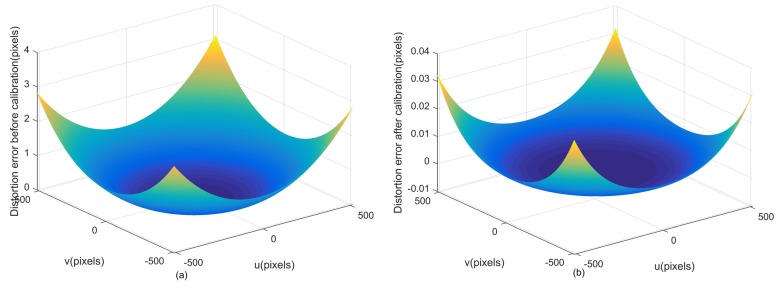
The lens distortion error. (**a**) The lens distortion error before calibration. (**b**) The lens distortion error after calibration.

Then, the proposed method is applied to the simulated data, and Figure 3 shows estimations of all systematic errors. The black and red lines indicate the estimation results and the preset true values of the calibration parameters, respectively. Specifically, Figure 3a shows estimations for the principal point $\left(u_{0},v_{0}\right)$ and the focal length *f*, Figure 3b for lens distortion coefficients $k_{1},k_{2},p_{1},p_{2}$, Figure 3c for the gyroscope bias and Figure 3d for fixed angle errors. The final calibration results are also listed in Table 3. It is shown that the estimations of all systematic errors are very close to the preset true values. The estimation error of the principal point is (0.0546,0.0334) pixel, the focal length error is 0.00002 mm, the gyroscope bias estimation errors are ${\left(0.0007,0.0002,0.0003\right)}^{\circ }$/h for the three gyroscopes, and the estimation errors of fixed angle errors are (1.8, 3.3, 0.8)” in three directions. The lens distortion error on the detector plane is shown in Figure 4. The distortion error at the edge of the detector is reduced from 3 pixels to 0.032 pixel when the proposed method is applied.

**Figure 5:**
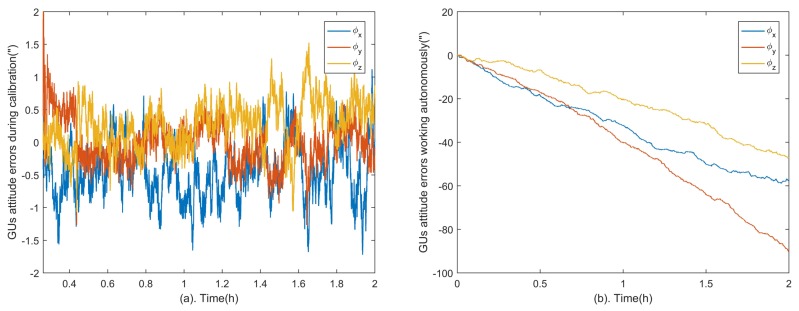
GUs attitude errors. Part (**a**) shows the GUs attitude errors in the calibration and the statistical results from time epoch 1 h to 2 h are $0.65^{\prime \prime },0.31^{\prime \prime },0.53^{\prime \prime }$ (RMS) in three axes. Part (**b**) shows attitude errors of the GUs without the aid of the star tracker for comparison.

In this calibration method, the role of the GUs is equivalent to the precision turntable of the laboratory-based calibrations. The GUs cannot keep high precision for a long time while working autonomously due to the cumulative error of gyroscopes. However, the GUs errors can be estimated and restrained by the star tracker through the proposed method. Figure 5a shows the attitude errors of the GUs in three axes. The convergent attitude accuracy is calculated from time epoch 1 h to 2 h, which are $0.65^{\prime \prime },0.31^{\prime \prime },0.53^{\prime \prime }$ (Root Mean Square, RMS) in x,y,z axes respectively. Compared to the attitude errors of GUs without the aid of the star tracker shown in Figure 5b, the cumulative error is eliminated and the accuracy is improved significantly. In other words, the GUs can serve as an accurate three-dimensional turntable in the calibration.

**Figure 6:**
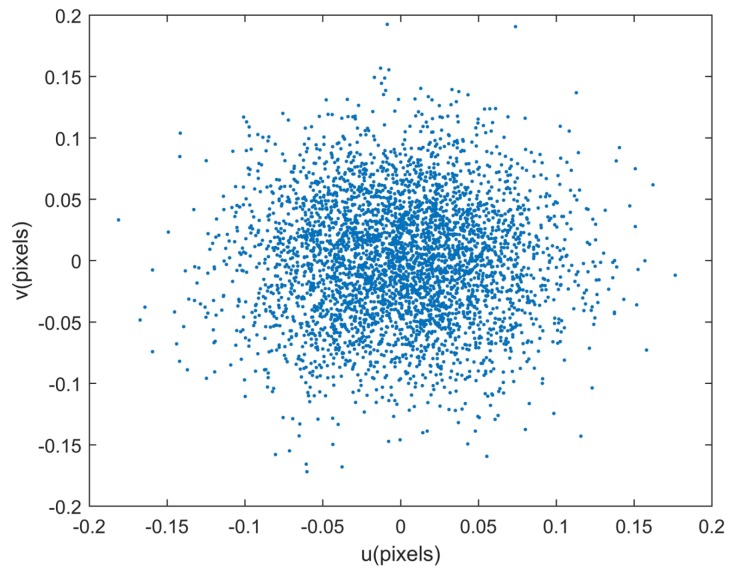
Reprojection errors in the Simulation. The statistical result is (0.05,0.05) pixel (RMS) in the two directions of the image plane.

**Figure 7:**
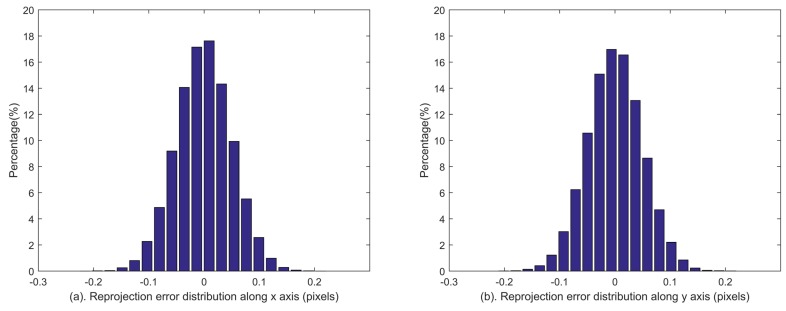
Distributions of reprojection errors along *x* (subfigure (**a**)) and *y* (subfigure (**b**)) axes in the simulation.

Given the estimated calibration parameters, the identified stars can be reprojected to the image plane. The reprojection errors [27], which are defined as the deviations between the reprojected star coordinates and the extracted ones, reflect the calibration accuracies of all systematic errors synthetically. The reprojection errors are calculated during the last 10 minutes of the simulation, which are shown in Figure 6. The distributions of reprojection errors are shown in Figure 7. The statistical reprojection accuracy is (0.05, 0.05) pixel (RMS) in the two directions of the image plane, which is almost the same as the preset centroid accuracy. It is shown that the reprojection errors induced by the systematic errors of the star tracker and GUs integrated system are remarkably reduced, which means the proposed method can achieve accurate calibration results.

### 3.2. Performance under Different Star Centroid Accuracies

The star centroid accuracy is one of the main factors affecting the calibration accuracy, and it differs under different noise levels of the detector. Simulations under different centroid accuracies are conducted in this section. The centroid accuracy is sequentially set to 0, 0.05, 0.10, 0.15, and 0.20 pixel, and other performance specifications of the star tracker and GUs are kept the same as in Section 3.1. For each star centroid accuracy, 100 random simulations are carried out. The calibration accuracy of each parameter is evaluated by the RMS value of estimation errors, and the statistical results are listed in Table 4.

From the statistical results under different centroid accuracies, it can be seen that the gyroscope bias estimations are scarcely affected by the centroid errors. Although the calibration accuracies of distortion coefficients are slightly affected, the maximum distortion can still be acceptably controlled below 0.035 pixel at the edge of the detector even when the centroid error reaches 0.20 pixel. Similarly, the calibration accuracy of the focus length can still reach 0.00027 mm when the centroid error reaches 0.20 pixel, which is accurate enough for the application of the integrated system. The influence of the centroid error mainly focuses on the estimations for the principal point $\left(u_{0},v_{0}\right)$ and subcomponents of fixed angles $\left(\Psi_{x},\Psi_{y}\right)$ due to the coupling between them, which will be analyzed in Section 3.4. Their estimation accuracies decline with increasing centroid error. Generally, the centroid accuracy of the star tracker is high enough (e.g., better than 0.10 pixel for the star tracker in our laboratory) during calibration, and high calibration accuracy can be acquired with the proposed method.

### 3.3. Performance under Different Gyroscope Noise Levels

Another factor affecting the calibration accuracy is the gyroscope noise (i.e., angular random walk). Simulations under different gyroscope noise levels are conducted in this section. The gyroscope bias is set to ${\left[0.1,0.1,0.1\right]}^{\circ }$/h, the angular random walk of the gyroscope is sequentially set to 0, 0.0001, 0.001, 0.01 and 0.1${}^{\circ }/\sqrt{h}$, and other performance specifications of the star tracker and GUs are the same as the simulation in Section 3.1. Similarly, for each gyroscope noise level, 100 random simulations are carried out. The statistical results are shown in Table 5.

It can be seen that the star tracker intrinsic parameters including the principal point $\left(u_{0},v_{0}\right)$, the focal length *f* and the distortion coefficients $\left(k_{1},k_{2},p_{1},p_{2}\right)$ can be accurately estimated, and are scarcely affected by the gyroscope noise when the noise level is below $0.1^{\circ }/\sqrt{h}$. The influence of the gyroscope noise mainly focuses on the estimations of the gyroscope bias and fixed angles. Although their calibration accuracies decline with increasing gyroscope noise level, the errors can be maintained at an acceptable level when the gyroscope angular random walk is below $0.01^{\circ }/\sqrt{h}$. Therefore, it shows a good prospect for the proposed method to be applied in the calibration of the integration of the star tracker and navigation grade gyroscopes.

### 3.4. Discussion on the Error Coupling

In Section 3.1, a typical star tracker and gyroscopes of navigation grade are simulated to verify the proposed method. The simulations with different star centroid accuracies and gyroscope noise levels demonstrate its robustness. The simulation results show that the estimation errors of the systematic errors mainly focus on the principal point and fixed angles. According to Table 4, the estimation accuracy of the principal point decreases from [0.007, 0.006] to [0.159, 0.182] pixel, and the accuracy of the fixed angles decreases from [0.5, 0.4, 0.4]” to [9.6, 8.3, 0.9]” when the centroid error varies from 0 to 0.20 pixel. According to Table 5, the estimation accuracies of the principal point and fixed angles cannot be further improved by increasing the gyroscope accuracy when the centroid accuracy is fixed. That is mainly because the principal point and fixed angles share similar physical characteristics and therefore strongly coupled. In this Section, the coupling between the principal point and fixed angles, which is the main problem of attitude-dependent calibration methods of the star tracker, will be discussed in theory.

The theoretical expression of the observation (δu,δv) with respect to the errors of the principal point and fixed angles is given by Equation (15). To simplify, only the terms containing the parameters of principal point and fixed angles are reserved, other terms are combined to be expressed by functions $g_{1}$ and $g_{2}$. In this way, Equation (15) can be further expressed as:

(27) $$\left\{\begin{array}{c} \delta u=\delta u_{0}+f\frac{x_{s}y_{s}}{z_{s}^{2}}\Psi_{x}-\left(f+f\frac{x_{s}^{2}}{z_{s}^{2}}\right)\Psi_{y}+f\frac{y_{s}}{z_{s}}\Psi_{z}+g_{1}\left(\delta f,\delta u_{d},\delta v_{d},\phi \right)\\ \delta v=\delta v_{0}+\left(f+f\frac{y_{s}^{2}}{z_{s}^{2}}\right)\Psi_{x}-f\frac{x_{s}y_{s}}{z_{s}^{2}}\Psi_{y}-f\frac{x_{s}}{z_{s}}\Psi_{z}+g_{2}\left(\delta f,\delta u_{d},\delta v_{d},\phi \right) \end{array}\right..$$

Take the star tracker in our laboratory as an example, the mean value of $z_{s}$ is approximately 16 times that of $x_{s}$ and $y_{s}$ for the stars evenly distributed in the image plane (i.e., $\left|\frac{{\bar{x}}_{s}}{{\bar{z}}_{s}}\right|,\left|\frac{{\bar{x}}_{s}}{{\bar{z}}_{s}}\right|\approx \frac{1}{16}$). The effects of terms $f\frac{x_{s}y_{s}}{z_{s}^{2}}\Psi_{x}$ and $f\frac{x_{s}^{2}}{z_{s}^{2}}\Psi_{y}$ in Equation (27) are smaller than that of the term $f\Psi_{y}$ by two orders of magnitude. If $\Psi_{y}=1^{\prime \prime }$, the change of the observation in Equation (27) caused by the term $f\frac{x_{s}^{2}}{z_{s}^{2}}\Psi_{y}$ is approximately 7.5 ×$10^{-5}$ pixel, which can be neglected compared to the effect caused by term $f\Psi_{y}$ (0.02 pixel). Therefore, $f\frac{x_{s}y_{s}}{z_{s}^{2}}\Psi_{x}$, $f\frac{x_{s}^{2}}{z_{s}^{2}}\Psi_{y}$,$f\frac{x_{s}y_{s}}{z_{s}^{2}}\Psi_{y}$ and $f\frac{y_{s}^{2}}{z_{s}^{2}}\Psi_{x}$ can be regarded as higher order terms. Neglecting these terms, Equation (27) can be approximated as:

(28) $$\left\{\begin{array}{c} \delta u\approx \delta u_{0}-f\Psi_{y}+f\frac{y_{s}}{z_{s}}\Psi_{z}+g_{1}\left(\delta f,\delta u_{d},\delta v_{d},\phi \right)\\ \delta v\approx \delta v_{0}+f\Psi_{x}-f\frac{x_{s}}{z_{s}}\Psi_{z}+g_{2}\left(\delta f,\delta u_{d},\delta v_{d},\phi \right) \end{array}\right..$$

According to Equation (28), the effects of subcomponents of fixed angle errors (i.e., $\Psi_{x}$ and $\Psi_{y}$) are constants (i.e., $f\Psi_{x}$ and $-f\Psi_{y}$ respectively), which are essentially the same as the effect of the error of principal point. Therefore, the estimation errors of the principal point and $(\Psi_{x}$, $\Psi_{y})$ are strongly coupled. The decoupling of $(\Psi_{x}$, $\Psi_{y})$ depends on the higher order terms in Equation (27), and the decoupling of $\Psi_{z}$ depends on the lower order terms $f\frac{y_{s}}{z_{s}}\Psi_{z}$ and $-f\frac{x_{s}}{z_{s}}\Psi_{z}$. This explains why $\Psi_{z}$ can be decoupled more accurately than $\Psi_{x}$ and $\Psi_{y}$ (See Table 4 and Table 5).

**Figure 8:**
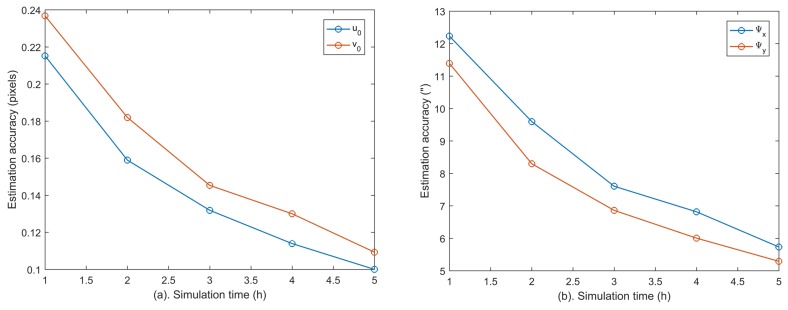
The calibration accuracies of $\left(u_{0},v_{0}\right)$ and ($\Psi_{x}$, $\Psi_{y}$) with extended calibration time when the centroid accuracy is 0.20 pixel.

According to Table 4, the estimation accuracies of the principal point and subcomponents of fixed angles $\left(\Psi_{x},\Psi_{y}\right)$ decrease with increasing centroid error due to the coupling between them. Their estimation accuracies drop to (0.159, 0.182) pixel and (9.6, 8.3)” within 2 h when the centroid accuracy is set to 0.20 pixel. To further decouple these errors, longer calibration time is needed. Therefore, we have extended the simulations to 5 h, and the estimation accuracies of $\left(u_{0},v_{0}\right)$ and ($\Psi_{x}$, $\Psi_{y}$) are calculated per hour as shown in Figure 8. It can be seen that their calibration accuracies can be further improved with longer calibration time.

**Figure 9:**
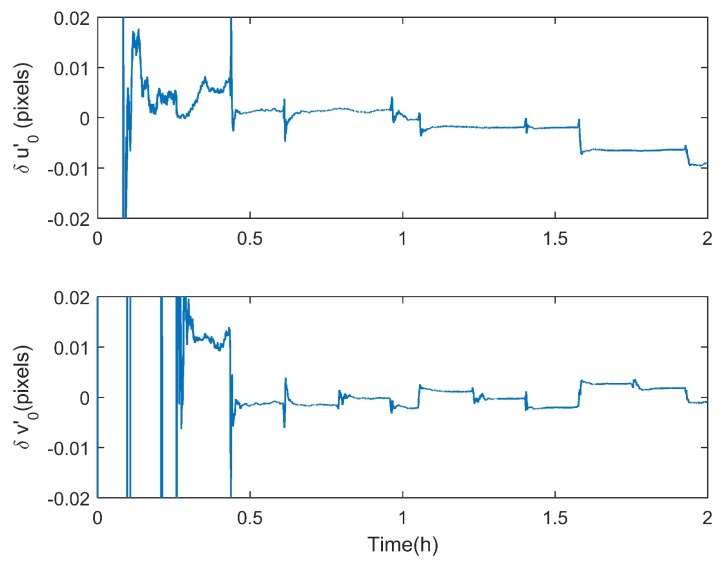
The estimation results of the equivalent principal point error $\left(\delta u_{0}^{\prime },\delta v_{0}^{\prime }\right)$ in the simulation, and the statistical result from time epoch 1 h to 2 h is (0.0039,0.0005) pixel (RMS).

Although the errors of the principal point and subcomponents of fixed angles $\left(\Psi_{x},\Psi_{y}\right)$ are strongly coupled due to the similarity of their physical characteristics, their comprehensive effects (i.e., $\left(\delta u_{0}-f\Psi_{y},\delta v_{0}+f\Psi_{x}\right)$) can be estimated quickly and accurately according to Equation (28). Since the effect of $\left(\delta u_{0}-f\Psi_{y},\delta v_{0}+f\Psi_{x}\right)$ is similar to that of the principal point, it is named as the equivalent principal point error $\left(\delta u_{0}^{\prime }=\delta u_{0}-f\Psi_{y},\delta v_{0}^{\prime }=\delta v_{0}+f\Psi_{x}\right)$. For a star tracker and GUs integrated system, it is the equivalent principal point error that affects its integrated attitude accuracy. For example, according to the simulation results in Section 3.1, the estimation errors of the principal point and fixed angles are both within arcsecond levels, which are (0.0546,0.0334) pixel and (1.8, 3.3, 0.8)” respectively. Fortunately, the attitude accuracy of the integrated system after calibration can reach the subarcsecond level, which are $0.65^{\prime \prime },0.31^{\prime \prime },0.53^{\prime \prime }$ (RMS) in x,y,z axes, respectively (See Figure 5a). Although $\left(u_{0},v_{0}\right)$ and ($\Psi_{x}$, $\Psi_{y}$) have not been fully decoupled, the estimations of the equivalent principal point error have already converged. Figure 9 shows the estimation results of the equivalent principal point error $\left(\delta u_{0}^{\prime },\delta v_{0}^{\prime }\right)$ of the simulation in Section 3.1, and the statistical result from time epoch 1 h to 2 h is (0.0039,0.0005) pixel (RMS). This explains why the attitude of the integrated system can be achieved accurately even though $\left(u_{0},v_{0}\right)$ and ($\Psi_{x}$, $\Psi_{y}$) have not been fully decoupled, which shows the advantage of the proposed method.

It is easy to understand that the coupling between $\left(u_{0},v_{0}\right)$ and $\left(\Psi_{x},\Psi_{y}\right)$ affects the decoupling accuracy between them, which can be improved by extending the calibration time. Moreover, the accuracy of the integrated system is mainly affected by the equivalent principal point error $\left(\delta u_{0}^{\prime },\delta v_{0}^{\prime }\right)$. Since $\left(\delta u_{0}^{\prime },\delta v_{0}^{\prime }\right)$ can converge quickly and accurately, the attitude accuracy of the integrated system is adequate for applications even though they have not been fully decoupled.

## 4. Experiment

**Figure 10:**
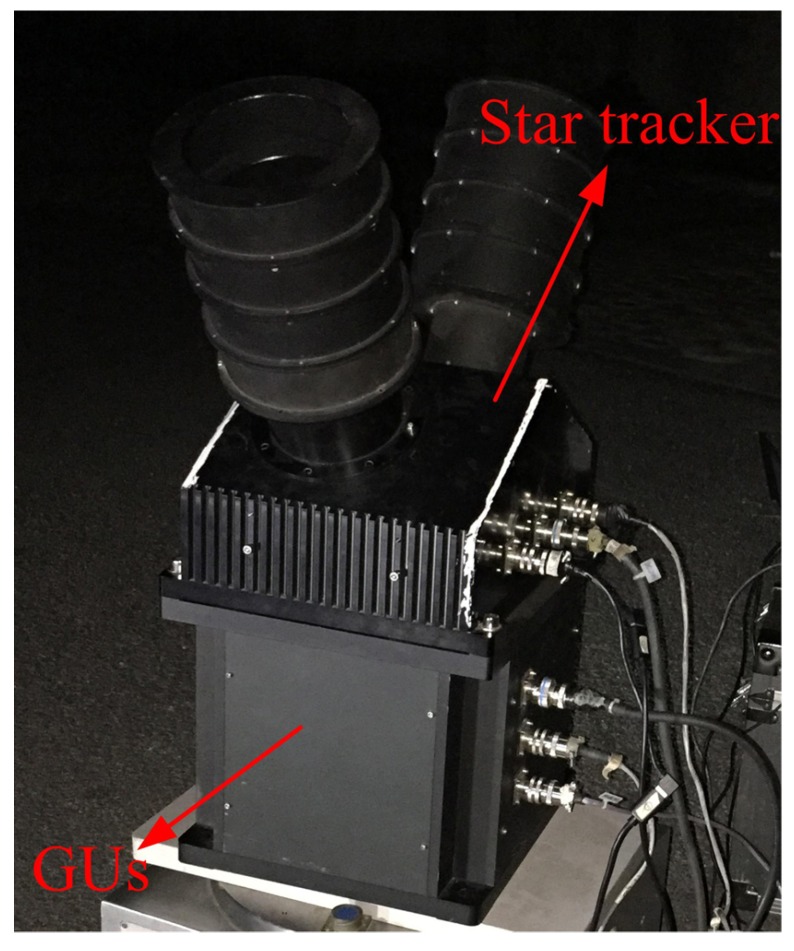
Experimental setup.

**Figure 11:**
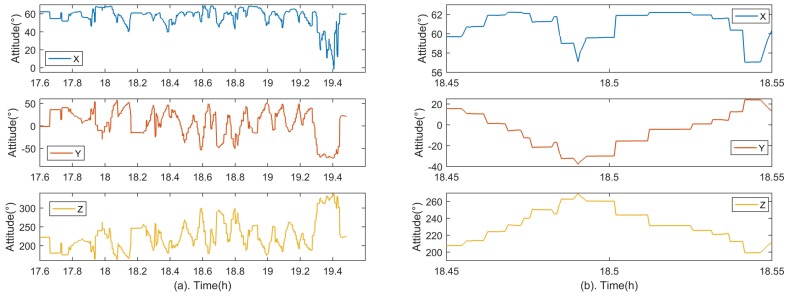
Attitude adjustments of the integrated system in the experiment. Part (**b**) is partial enlarged detail of (**a**) in order to display fine details.

**Figure 12:**
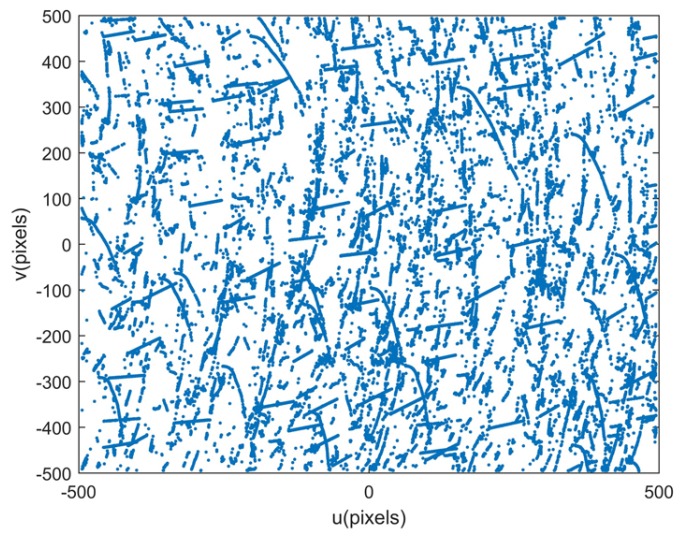
The distribution of all sampled stars over the image plane.

Experiments were conducted to verify the proposed method at Hengshan National Forest Park (Hengyang, China). The experimental setup is shown in Figure 10. The star tracker and GUs are fixed together, and their performance specifications are consistent with the previous simulation in Section 3.1. Similarly, the attitude adjustments for the integrated system are essential during the experiment shown in Figure 11a. As shown in Figure 11b, the attitude of the system is adjusted gradually and maintained at each position so that stars can be evenly distributed over the image plane, which is beneficial for the lens distortion calibration. The distribution of all sampled stars during the experiment is shown in Figure 12.

### 4.1. Estimation of Systematic Errors

**Figure 13:**
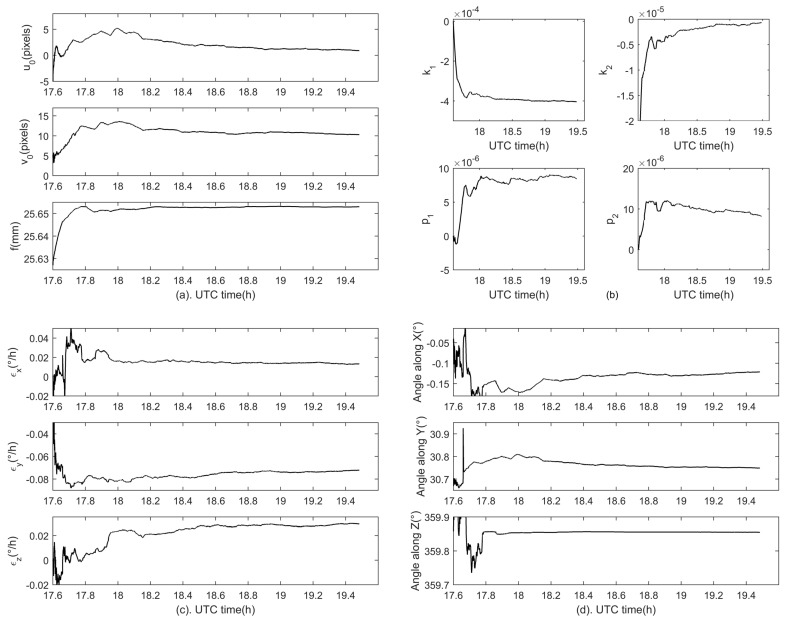
Estimation results of all systematic errors in the experiment. (**a**) shows estimations for the principal point $\left(u_{0},v_{0}\right)$ and the focal length *f*, (**b**) shows estimations for lens distortion coefficients $\left(k_{1},k_{2},p_{1},p_{2}\right)$, (**c**,**d**) show estimations for the gyroscope bias and fixed angles respectively.

The systematic errors including the star tracker intrinsic parameter errors, GUs errors and fixed angle errors are estimated with the proposed method, and the estimation results are shown in Figure 13. Specifically, Figure 13a shows the estimations for the principal point $\left(u_{0},v_{0}\right)$ and the focal length *f*, Figure 13b for lens distortion coefficients $\left(k_{1},k_{2},p_{1},p_{2}\right)$, Figure 13c for the gyroscope bias and Figure 13d for fixed angles. It can be seen that the convergence time of all the estimated parameters is less than an hour. The final estimations for the star tracker intrinsic parameters are listed in Table 6, and estimations for fixed angles are given in Table 7.

### 4.2. Performance Evaluation

**Figure 14:**
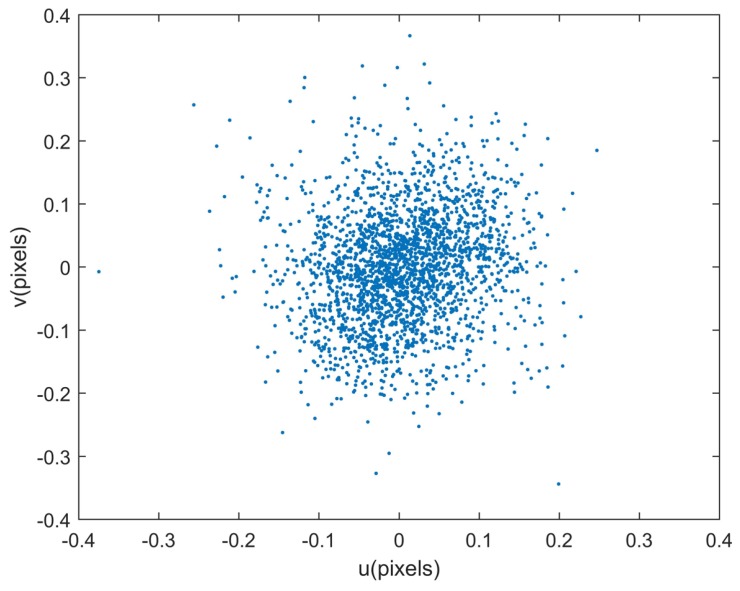
Reprojection errors after calibration, and statistical results in two directions are 0.0732 pixel and 0.0909 pixel respectively.

**Figure 15:**
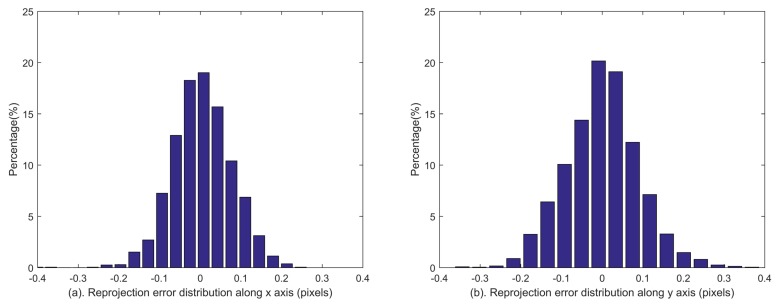
Distributions of reprojection errors along *x* (subfigure (**a**)) and *y* (subfigure (**b**)) axes in the experiment.

Since the true values of all calibrated parameters are unknown, the calibration accuracy of each parameter cannot be calculated directly in the experiment. Therefore, the reprojection error is used to evaluate the calibration accuracy synthetically. A supplemental experiment is conducted to evaluate the performance of the calibration method. The reprojection errors are shown in Figure 14, and statistical results in two directions are 0.0732 pixel and 0.0909 pixel respectively. The distributions of reprojection errors are shown in Figure 15. Synthetically, the calibration accuracy is (0.0732, 0.0909) pixel expressed in the form of the star reprojection error.

## 5. Conclusions

We have shown that a star tracker and GUs can be used together to take full advantage of the benefits of each. Existing calibration methods mainly focus on the single star tracker, and separate calibrations of the star tracker and GUs increase the complexity of the calibration process. To optimally estimate the systematic errors (i.e., the star tracker intrinsic parameter errors, GUs errors and fixed angle errors between them) of the star tracker and GUs integrated system from a global perspective, we propose a comprehensive calibration method for this integrated system by observing the PSCE in the image plane, and all systematic errors can be estimated simultaneously. Simulations were designed to validate the proposed method, and results show that all estimations converge to the preset true values. Simulations under different star centroid accuracies indicate that the calibration accuracies of the focal length, the lens distortion, the gyroscope bias and subcomponent of the fixed angle errors along *z* axis are scarcely affected. Although the calibration accuracies of the principal point and subcomponents of fixed angle errors along *x* and *y* axes (i.e., $\Psi_{x}$ and $\Psi_{y}$) decline with increasing centroid error, their calibration accuracies can be further improved by extending the calibration time. Simulations under different gyroscope noise levels indicate that the proposed method can be applied to the calibration of the integration of the star tracker and navigation grade gyroscopes. The coupling between the errors of the principal point and subcomponents of the fixed angles (i.e., $\Psi_{x}$ and $\Psi_{y}$) is analysed in theory. Results show that they are strongly coupled due to the similarity of their physical characteristics. For a star tracker and GUs integrated system, it is the equivalent principal point error $\left(\delta u_{0}^{\prime }=\delta u_{0}-f\Psi_{y},\delta v_{0}^{\prime }=\delta v_{0}+f\Psi_{x}\right)$ that affects its integrated attitude accuracy. Since $\left(\delta u_{0}^{\prime },\delta v_{0}^{\prime }\right)$ can converge quickly and accurately in the simulation, the attitude accuracy of the integrated system is adequate for applications even though they have not been fully decoupled. Experiments of nightsky observations were conducted and the systematic errors of the integrated system were successfully estimated with the proposed method. Considering that the calibration accuracy cannot be calculated directly due to the lack of true model parameters, we adopt the reprojection error induced by errors of all estimated parameters to evaluate the calibration accuracy synthetically, which are 0.0732 pixel and 0.0909 pixel in two directions respectively. Therefore, the proposed method has great potential to be used in the calibration of the star tracker and GUs integrated system.

## Figures and Tables

**Table 1:** Simulation parameters of the star tracker and GUs.

Parameter	Value	Parameter	Value
Active pixels	1024×1024	Exposure period	180 ms
Pixel pitch	6.45 × 6.45μm${}^{2}$	Star tracker update frequency	2 Hz
Principal point	(5,10) pixels	Focal length	25.6 mm
Distortion coefficients $\left(k_{1},k_{2}\right)$	(2 × $10^{-4}$,−1 × $10^{-6}$)	Distortion coefficients $\left(p_{1},p_{2}\right)$	(2 × $10^{-5}$,2 × $10^{-5}$)
Star centroid accuracy	(0.05,0.05) pixel	Gyroscope sampling frequency	100 Hz
Gyroscope angular random walk	$0.001^{\circ }/\sqrt{h}$	Gyroscope bias	${\left[0.01,0.01,0.01\right]}^{\circ }$/h
True fixed angles	${\left[-0.1,30.0,359.8\right]}^{\circ }$	Fixed angle errors	${\left[100,200,300\right]}^{\prime \prime }$

**Table 2:** The rotation sequence of the integrated system for calibration.

Number	Rotation Axis	Rotation Angle (${}^{\circ }$)
1	$X_{g}$	30
2	$Y_{g}$	30
3	$Z_{g}$	30
4	$Z_{g}$	−30
5	$Y_{g}$	−30
6	$X_{g}$	−30

**Table 3:** Estimation results of systematic errors in the simulation.

Parameter	True	Estimation	Estimation Error	Parameter	True	Estimation	Estimation Error
$u_{0}$ (pixels)	5	5.0546	0.0546	$\varepsilon_{x}$ (${}^{\circ }$/h)	0.01	0.0093	0.0007
$v_{0}$ (pixels)	10	10.0334	0.0334	$\varepsilon_{y}$ (${}^{\circ }$/h)	0.01	0.0102	0.0002
*f* (mm)	25.6	25.60002	0.00002	$\varepsilon_{z}$ (${}^{\circ }$/h)	0.01	0.0097	0.0003
$k_{1}$	2 × $10^{-4}$	1.9960 × $10^{-4}$	0.0040 × $10^{-4}$	$\Psi_{x}\left({}^{\prime \prime }\right)$	100	101.8	1.8
$k_{2}$	−1 ×$10^{-6}$	−1.0737 ×$10^{-6}$	0.0737 × $10^{-6}$	$\Psi_{y}\left({}^{\prime \prime }\right)$	200	196.7	3.3
$p_{1}$	2 × $10^{-5}$	2.0041 × $10^{-5}$	0.0041 × $10^{-5}$	$\Psi_{z}\left({}^{\prime \prime }\right)$	300	299.2	0.8
$p_{2}$	2 × $10^{-5}$	1.9942 × $10^{-5}$	0.0058 × $10^{-5}$				

**Table 4:** Calibration accuracies of all systematic errors under different centroid accuracies.

Centroid Accuracy (Pixels)	0	0.05	0.10	0.15	0.20
$u_{0}$ (pixels)	0.007	0.041	0.076	0.112	0.159
$v_{0}$ (pixels)	0.006	0.045	0.083	0.120	0.182
*f* (mm)	2.6 × $10^{-5}$	3.0$\times 10^{-5}$	7.3 × $10^{-5}$	1.5 × $10^{-4}$	2.7 × $10^{-4}$
$k_{1}$	4.3 × $10^{-7}$	3.7 × $10^{-7}$	4.5 × $10^{-7}$	8.8 × $10^{-7}$	1.7 × $10^{-6}$
$k_{2}$	7.2 × $10^{-8}$	7.7 × $10^{-8}$	9.4 × $10^{-8}$	1.1 × $10^{-7}$	1.5 × $10^{-7}$
$p_{1}$	3.1 × $10^{-7}$	3.7 × $10^{-7}$	4.8 × $10^{-7}$	6.3 × $10^{-7}$	8.7 × $10^{-7}$
$p_{2}$	2.9 × $10^{-7}$	3.4 × $10^{-7}$	4.8 × $10^{-7}$	6.3 × $10^{-7}$	9.4 × $10^{-7}$
$\varepsilon_{x}$ (${}^{\circ }$/h)	3.2 × $10^{-4}$	3.8 × $10^{-4}$	3.6 × $10^{-4}$	3.5 × $10^{-4}$	3.7 × $10^{-4}$
$\varepsilon_{y}$ (${}^{\circ }$/h)	3.4 × $10^{-4}$	3.4 × $10^{-4}$	3.5 × $10^{-4}$	3.4 × $10^{-4}$	3.3 × $10^{-4}$
$\varepsilon_{z}$ (${}^{\circ }$/h)	3.8 × $10^{-4}$	3.9 × $10^{-4}$	3.5 × $10^{-4}$	3.7 × $10^{-4}$	3.8 × $10^{-4}$
$\Psi_{x}\left({}^{\prime \prime }\right)$	0.5	2.4	4.4	6.3	9.6
$\Psi_{y}\left({}^{\prime \prime }\right)$	0.4	2.1	3.9	5.8	8.3
$\Psi_{z}\left({}^{\prime \prime }\right)$	0.4	0.5	0.6	0.9	0.9

**Table 5:** Calibration accuracies of all systematic errors under different gyroscope noise levels.

Angular Random Walk (${}^{\circ }/\sqrt{h}$)	0	0.0001	0.001	0.01	0.1
$u_{0}$ (pixels)	0.042	0.042	0.045	0.044	0.045
$v_{0}$ (pixels)	0.044	0.045	0.043	0.040	0.040
*f* (mm)	2.8 × $10^{-5}$	2.8 × $10^{-5}$	3.2 × $10^{-5}$	2.7 × $10^{-5}$	2.7$\times 10^{-5}$
$k_{1}$	3.8 × $10^{-7}$	3.8 × $10^{-7}$	3.9 × $10^{-7}$	3.7 × $10^{-7}$	3.6$\times 10^{-7}$
$k_{2}$	7.7 × $10^{-8}$	7.6 × $10^{-8}$	7.7 × $10^{-8}$	7.7 × $10^{-8}$	7.7$\times 10^{-8}$
$p_{1}$	3.9 × $10^{-7}$	3.7 × $10^{-7}$	3.7 × $10^{-7}$	3.8 × $10^{-7}$	3.9$\times 10^{-7}$
$p_{2}$	3.6 × $10^{-7}$	3.6 × $10^{-7}$	3.5 × $10^{-7}$	3.5 × $10^{-7}$	3.4$\times 10^{-7}$
$\varepsilon_{x}$ (${}^{\circ }$/h)	3.0 × $10^{-5}$	7.0 × $10^{-5}$	7.7 × $10^{-4}$	6.8 × $10^{-3}$	5.4 × $10^{-2}$
$\varepsilon_{y}$ (${}^{\circ }$/h)	4.2 × 0-5	7.9 × $10^{-5}$	8.3 × $10^{-4}$	6.9$\times 10^{-3}$	5.4$\times 10^{-2}$
$\varepsilon_{z}$ (${}^{\circ }$/h)	3.7 × $10^{-5}$	8.3 × $10^{-5}$	6.9 × $10^{-4}$	7.2 × $10^{-3}$	4.9 × $10^{-2}$
$\Psi_{x}\left({}^{\prime \prime }\right)$	2.4	2.4	2.3	3.1	22.2
$\Psi_{y}\left({}^{\prime \prime }\right)$	2.2	2.2	2.3	3.6	22.5
$\Psi_{z}\left({}^{\prime \prime }\right)$	0.7	0.7	0.6	2.0	19.9

**Table 6:** Estimation results of the star tracker intrinsic parameters in the experiment.

$u_{0}$ (Pixels)	$v_{0}$ (Pixels)	*f* (mm)	$k_{1}$	$k_{2}$	$p_{1}$	$p_{2}$
0.9346	10.3034	25.6530	−4.0411 ×$10^{-4}$	−6.2924 ×$10^{-7}$	8.4867 × $10^{-6}$	8.1853 × $10^{-6}$

**Table 7:** Estimation results of fixed angles in the experiment.

Component	*X*	*Y*	*Z*
Result(${}^{\circ }$)	−0.1210	30.7491	359.8555

## Abbreviations

The following abbreviations are used in this manuscript:

GUs	Gyroscope units
PSC	Predicted star centroid
ESC	Extracted star centroid
PSCE	Predicted star centroid error
